# The 3′ UTR Pseudoknot-Containing Region of Arteriviruses Acts as a PAMP to Activate RIG-I- and TLR8-Dependent Type I Interferon Responses

**DOI:** 10.3390/v18101117

**Published:** 2026-10-09

**Authors:** Sha Xie, Lu Zhang, Yan-Gang Sun, Meixin Shu

**Affiliations:** 1 School of Medicine, Henan University of Chinese Medicine, No.156 Jinshui East Road, Zhengdong New District, Zhengzhou 450046, China; 2 Engineering Technology Research Center of TCM Health Industry in Henan Province, No.156 Jinshui East Road, Zhengdong New District, Zhengzhou 450046, China; 3 Engineering Research Center of TCM Health Management and Application in Henan Province, No.156 Jinshui East Road, Zhengdong New District, Zhengzhou 450046, China

**Keywords:** arteriviruses, 3′ UTR pseudoknot, RIG-I, TLR8, type I interferon

## Abstract

Porcine reproductive and respiratory syndrome virus (PRRSV) and other members of the Arteriviridae family cause substantial economic losses in livestock. However, their pathogen-associated molecular patterns (PAMPs) remain poorly characterized. Here, we investigated whether the 3′ untranslated region (UTR) pseudoknot structures of Arteriviruses act as PAMPs and explored the underlying innate immune recognition mechanisms. Bioinformatics predictions (RNAalifold, RNArtist, and RNAComposer) revealed that the 3′ UTR pseudoknot structures are conserved across all four Arteriviruses. In vitro transcribed pseudoknot RNAs significantly upregulated the mRNA expression of type I interferons (IFNα/β) and interferon-stimulated genes (protein kinase R, PKR; interferon-stimulated gene 15, ISG15; interferon-stimulated gene 56, ISG56) in Marc145 cells. Screening of RNA-sensing pattern recognition receptors (retinoic acid-inducible gene I, RIG-I; melanoma differentiation-associated protein 5, MDA5; Toll-like receptor 3/7/8/9, TLR3/7/8/9) in pseudoknot RNA-stimulated cells showed that RIG-I and TLR8 were significantly upregulated, whereas MDA5, TLR3, TLR7, and TLR9 were not. Short hairpin RNA (shRNA)-mediated knockdown of RIG-I or TLR8 significantly reduced pseudoknot-induced expression of IFNβ, PKR, and ISG56, confirming that both receptors are functionally required for this response. Upregulation of myeloid differentiation primary response 88 (MyD88), interferon regulatory factor 7 (IRF7), and signal transducer and activator of transcription 2 (STAT2) suggested that IFNα/β and downstream interferon-stimulated genes (ISGs) were induced via the TLR8-MyD88-IRF7 and RIG-I-IRF7 signaling pathways. Moreover, small-angle X-ray scattering (SAXS) analysis of the PRRSV pseudoknot RNA complexed with the porcine RIG-I regulatory domain was consistent with the formation of a complex between the pseudoknot RNA and RIG-I. In conclusion, this study identified the 3′ UTR pseudoknot-containing region of Arteriviruses as a conserved PAMP that activates RIG-I- and TLR8-dependent type I IFN responses, providing a mechanistic basis and potential targets for antiviral therapy and vaccine adjuvant development against Arterivirus infections.

## 1. Introduction

The *Arteriviridae* family comprises equine arteritis virus (EAV), porcine reproductive and respiratory syndrome virus (PRRSV), lactate dehydrogenase-elevating virus (LDV), simian hemorrhagic fever virus (SHFV), and wobbly possum disease virus (WPDV) [1]. Arterivirus infections cause severe diseases in livestock, including equine arteritis virus (EAV), porcine reproductive and respiratory syndrome (PRRS), and highly pathogenic PRRS (HP-PRRS), resulting in substantial economic losses [2,3,4]. PRRSV remains a major pathogen in the Chinese pig industry, with recent epidemiological data indicating widespread circulation and considerable economic losses. [5,6,7].

Arteriviruses are single-stranded positive-sense RNA viruses with a genome of 12.7–15.7 kb, a 5′ cap and a 3′ polyA tail. The genome contains 5′ and 3′ untranslated regions (UTRs) and 9–12 open reading frames (ORFs). ORF1a and ORF1b encode two polyproteins (pp1a and pp1ab) that are processed into multiple non-structural proteins (NSPs); downstream ORFs encode structural proteins such as E, M and N [8]. Arteriviruses use discontinuous transcription to generate subgenomic mRNAs, which share a common 3′ UTR and a common 5′ UTR leader sequence containing important transcription regulatory sequences (TRSs) [9]. During replication, the two stem-loop structures (SL) at the 3′ UTR interact to form a pseudoknot structure that is highly conserved among Arteriviruses and acts as a molecular switch regulating negative-strand RNA synthesis [10]. The upstream stem-loop (SL1) of this pseudoknot can also form a “kissing interaction” with a hairpin in the N protein gene, which is required for RNA replication [11].

Regarding innate immune recognition of Arteriviruses, previous studies have mainly focused on pattern recognition receptor (PRR) identification. LDV infection activates the Toll-like receptor 7 (TLR7) pathway to induce type I interferon (IFN) [12]; PRRSV can modulate TLR3 and TLR7/9 expression [13] and also trigger the TLR4 pathway to produce inflammatory cytokines [14]. After PRRSV enters host cells via receptor-mediated endocytosis, its genomic RNA activates the RIG-I and TLR3 signaling pathway [15]. However, the pathogen-associated molecular patterns (PAMPs) of Arteriviruses are not well characterized. Our previous work demonstrated that the pseudoknot structure in the PRRSV 3′ UTR acts as a PAMP [15]. Sequence alignment revealed that the pseudoknot is highly conserved among EAV, LDV, SHFV and PRRSV, and secondary structure prediction indicated that all four viruses possess a conserved pseudoknot interaction region.

In this study, we investigated whether the 3′ UTR pseudoknot structures of different Arteriviruses represent a new class of PAMP molecules and explored the underlying innate immune recognition mechanism. Using bioinformatic prediction, in vitro transcribed pseudoknot RNAs, quantitative real-time PCR (qRT-PCR), short hairpin RNA (shRNA) knockdown, recombinant protein expression, and small-angle X-ray scattering (SAXS) structural analysis, we showed that the conserved pseudoknots of EAV, PRRSV, LDV and SHFV were involved in host RIG-I and TLR8, activated type I IFN responses via both the RIG-I and TLR8 signaling pathways, and subsequently induced ISG expression. These findings provide a theoretical basis for the prevention and control of Arterivirus infections.

## 2. Materials and Methods

### 2.1. Bioinformatic Prediction of Pseudoknot Structures

The secondary structures of the 3′ UTR pseudoknot RNAs from PRRSV (strain BJ4), EAV, SHFV and LDV were predicted using the RNAalifold web server (http://rna.tbi.univie.ac.at/cgi-bin/RNAWebSuite/RNAalifold.cgi, accessed on 5 May 2024). Predictions were based on minimum free energy with default parameters (37 °C, 1 M NaCl, no divalent ions, no maximum distance constraint for base pairing). Secondary structures were visualized and edited using RNArtist and used for tertiary structure prediction with the RNAComposer web server (http://rnacomposer.ibch.poznan.pl/). The resulting protein data bank (PDB) files were visualized and processed using PyMOL 3.0.2.

### 2.2. In Vitro Transcription and Purification of Pseudoknot RNAs

DNA templates encoding the 3′ UTR pseudoknot sequences of PRRSV-BJ4, EAV, SHFV and LDV were designed based on the conserved pseudoknot structures (Table 1). Complementary single-stranded DNA oligonucleotides were synthesized (Shanghai Bioengineering Co., Ltd., Shanghai, China) and annealed using a DNA oligonucleotide annealing buffer kit (TaKaRa, Takara Biotechnology (Dalian) Co., Ltd., Dalian, China) following the manufacturer’s instructions. Capped pseudoknot RNAs were synthesized using the mMESSAGE mMACHINE™ T7 Transcription Kit (AM1344, Invitrogen, Carlsbad, CA, USA) according to the manufacturer’s protocol. For the positive control 3pRNA (3RP), the MEGAshortscript™ T7 Transcription Kit (AM1354, Invitrogen) was used. Both transcription reactions were incubated at 37 °C for 6 h, followed by the addition of 1 μL of TURBO DNase (provided in the kits) and incubation at 37 °C for 15 min to remove the DNA template.

The RNA products were purified using the Monarch^®^ RNA Cleanup Kit (NEB, Ipswich, MA, USA) according to the manufacturer’s instructions. Briefly, the RNA sample was mixed with 100 μL of Binding Buffer, 30 μL of DNase/RNase-Free Water and 150 μL of absolute ethanol, and then transferred to a purification column. After centrifugation at 10,000 rpm for 1 min, the column was washed twice with 500 μL of wash buffer, centrifuged at 10,000 rpm for 1 min to remove residual ethanol, and air-dried. Purified RNA was eluted with 20 μL of DNase/RNase-Free water, incubated at room temperature for 5 min, and recovered by centrifugation at 10,000 rpm for 1 min. The eluate was reloaded and centrifuged again to maximize recovery. RNA concentration was measured using a NanoDrop spectrophotometer, and samples were aliquoted, labeled, and stored at −80 °C.

### 2.3. Cell Culture and Transfection

Marc145 cells were cultured in Dulbecco’s Modified Eagle Medium (DMEM) supplemented with 10% fetal bovine serum and 1% penicillin-streptomycin at 37 °C in a 5% CO_2_ incubator. Cells were routinely passaged upon reaching confluence using trypsin-EDTA. For transfection, cells were seeded in 48-well plates at 40,000–50,000 cells per well and cultured to 80–90% confluence. Pseudoknot RNAs (0.5, 0.75, or 1 μg) were transfected using the TransMessenger Transfection Reagent (QIAGEN, Venlo, The Netherlands) according to the manufacturer’s protocol. In brief, RNA was mixed with Enhancer R in Buffer EC, incubated for 5 min at room temperature, then combined with the TransMessenger Reagent pre-diluted in Buffer EC and incubated for another 10 min to form transfection complexes. The complexes were diluted in Opti-MEM, added dropwise to the cells, and incubated for 2 h. After removal of the complexes, cells were rinsed with PBS and cultured in fresh complete medium for an additional 10 h. Polyinosinic: polycytidylic acid (poly(I:C) (InvivoGen, San Diego, CA, USA)) and 3RP were used as positive controls at the same concentrations. Mock-transfected cells received only the transfection reagent.

### 2.4. shRNA Knockdown of RIG-I and TLR8

shRNA sequences targeting monkey RIG-I and TLR8 were designed using the siRNA Wizard online tool (InvivoGen). The selected 19 bp sequences containing at least two G-C pairs were incorporated into forward and reverse oligonucleotides and verified for hairpin structures (Table 2). The oligonucleotides were annealed using a gradient cooling program and ligated into the pLVX-shRNA1 vector (Clontech, Mountain View, CA, USA) via BamHI and EcoRI sites. The ligation products were transformed into competent cells, and positive clones were selected on ampicillin-containing LB plates. Plasmids were extracted, verified by sequencing, and then transfected into Marc145 cells using jetPRIME^®^ reagent (Polyplus, Illkirch-Graffenstaden, France) at a DNA:reagent ratio of 1:1. After 48 h, knockdown efficiency was confirmed by qRT-PCR. The efficiency of each shRNA was calculated as the percentage reduction in target mRNA relative to cells transfected with control shRNA, and shRNAs achieving > 60% reduction were used for subsequent experiments. For the knockdown experiment, shRNA-expressing Marc145 cells were transfected with the optimal concentration of each pseudoknot RNA (determined in preliminary experiments), and cells were harvested at 12 h post-transfection for qRT-PCR analysis.

### 2.5. RNA Extraction and Quantitative Real-Time PCR (qRT-PCR)

Total RNA was extracted from transfected Marc145 cells using TRIzol reagent (Thermo Fisher Scientific, Waltham, MA, USA) according to the manufacturer’s protocol. First-strand cDNA was synthesized from 1 μg of total RNA using the PrimeScript RT Kit (TaKaRa) with random primers. qRT-PCR was performed using SYBR Green PCR Master Mix (Roche, Basel, Switzerland) on a Real-Time PCR System QS7 (Thermo Fisher Scientific) under the following conditions: 95 °C for 60 s, followed by 40 cycles of 95 °C for 15 s and 55 °C for 34 s. The primer sequences used for detection of IFNα, IFNβ, PKR, ISG15, ISG56, RIG-I, MDA5, TLR3, TLR7, TLR8, TLR9, MyD88, MAPK1, MAPK8, NEMO, DDX3X, IKKα, IKKβ, MAVS, p50, p65, AP-1, TBK1, IKKε, IRF3, IRF7, TYK2, JAK1, STAT1, STAT2, STAT3, STAT4, STAT5, STAT6, IRF9, MX1, OAS1a, and the housekeeping gene glyceraldehyde-3-phosphate dehydrogenase (GAPDH) are listed in Table 3. The relative expression levels were calculated using the 2^−ΔΔCT^ method after normalization to GAPDH.

### 2.6. Complex Formation and Small-Angle X-Ray Scattering (SAXS) Analysis

The coding sequence of the porcine RIG-I regulatory domain (RD, amino acids 806–943) was amplified from porcine alveolar macrophages (PAMs) cDNA and cloned into a pE-SUMO vector (Novoprotein, Suzhou, China). The recombinant plasmid was transformed into *E. coli* Transetta (DE3). Protein expression was induced with 0.2 mM isopropyl β-D-1-thiogalactopyranoside (IPTG) at 18 °C for 16 h. The soluble fraction was purified by Ni-affinity chromatography (HisTrap excel, Cytiva, Marlborough, MA, USA), followed by cation exchange (HiTrap SP HP, Cytiva, Marlborough, MA, USA). After small ubiquitin-like modifier (SUMO) tag cleavage, the protein was re-purified by a second Ni-affinity and cation exchange step, and finally subjected to gel filtration on a Superdex 200 Increase 10/300 GL column. Purity was assessed by SDS-PAGE and Western blot [15].

The PRRSV 3′ UTR pseudoknot RNA and purified pRIG-I-RD were mixed at a 1:2 molar ratio, incubated on ice for 1 h, and purified by gel filtration on a Superdex 200 Increase 10/300 GL column (Cytiva, Marlborough, MA, USA) equilibrated with 20 mM Tris-HCl pH 8.0 and 150 mM NaCl. The complex was concentrated to 1–5 mg/mL for SAXS analysis. SAXS data were collected at beamline BL19U2 of the Shanghai Synchrotron Radiation Facility (SSRF). The complex was prepared at 1, 3, and 5 mg/mL in the same buffer. For each sample, data were collected in the order of buffer, low-concentration sample, high-concentration sample, and buffer again. Each sample was exposed 10 times (1 s per frame) and averaged to improve the signal-to-noise ratio. Buffer scattering was subtracted. The scattering curves at 1 and 5 mg/mL overlapped well after scaling, confirming data quality.

Data processing was performed with the ATSAS 3.3.0 package. Guinier analysis yielded a radius of gyration (Rg) of 36.19 Å, an sRg range of 0.73–1.3, and zero-angle scattering intensity (I_0_) = 18.11. The sRg value below 1.3 indicated high-quality data suitable for further analysis. GNOM analysis gave a maximum dimension (Dmax) of 161.47 Å, with good agreement between experimental and theoretical curves. The pair distance distribution function (P(*r*)) curve showed two peaks, suggesting that the complex is an elongated macromolecular structure with multiple domains and at least two centers. Ab initio shape reconstruction was performed using DAMMIF, and models were fitted into the SAXS envelope using Chimera.

### 2.7. Statistical Analysis

All experiments were performed with at least three independent biological replicates. Data are presented as mean ± standard deviation (SD). Statistical significance was assessed using one-way analysis of variance (ANOVA) followed by Dunnett’s multiple comparisons test for multiple comparisons in GraphPad Prism 8. Differences were considered significant when *p* < 0.05.

## 3. Results

### 3.1. Structural Prediction of the 3′ UTR Pseudoknot in Arteriviridae Members

The secondary structures of the 3′ UTR pseudoknot RNAs from PRRSV (strain BJ4), EAV, SHFV and LDV were predicted using the RNAalifold web server based on minimum free energy with default parameters. The predicted secondary structures were further edited using RNArtist. Tertiary structures were then predicted based on the secondary structures using RNAComposer, and the resulting PDB files were visualized with PyMOL. The results demonstrated that all four viruses contain a conserved pseudoknot structure. As shown in Figure 1, the predicted secondary structures and tertiary conformations were presented for PRRSV-BJ4 (A), EAV (B), SHFV (C), and LDV (D). Interactions between loop structures are indicated by grey lines.

### 3.2. Upregulation of Type I IFN and ISGs mRNA Expression by Arterivirus Pseudoknot RNAs

Marc145 cells were transfected with 0.5, 0.75, or 1 μg of in vitro transcribed 3′UTR pseudoknot RNAs from PRRSV-BJ4, SHFV, EAV, and LDV. At 12 h post-transfection, qRT-PCR was used to measure mRNA levels of type I interferons (IFNα/β) and interferon-stimulated genes (PKR, ISG15, ISG56). Poly(I:C) and 3RP served as positive controls, and all values were normalized to mock-transfected controls.

As shown in Figure 2A,B, all four pseudoknot RNAs induced upregulation of IFNβ, with the EAV and LDV pseudoknots eliciting the strongest responses (up to 362-fold and 422-fold, respectively). The PRRSV-BJ4 and SHFV pseudoknots also significantly increased IFNβ expression (up to 213-fold and 38-fold, respectively). In contrast, IFNα was modestly induced (less than 3-fold) by all pseudoknots. The positive controls 3RP and poly(I:C) induced robust IFNβ expression (up to 1395.7-fold and 1516-fold, respectively), confirming the validity of the experimental system.

Regarding ISG expression (Figure 2C–E), all pseudoknots markedly upregulated PKR, ISG15, and ISG56. Notably, the magnitude of ISG56 induction varied considerably among the four viruses: EAV and LDV induced extremely high levels (up to 1106.8-fold and 1145-fold, respectively), whereas PRRSV-BJ4 and SHFV induced more moderate but still substantial increases (up to 271.1-fold and 173.2-fold, respectively). PKR was induced approximately 4- to 5-fold by most pseudoknots, and ISG15 was induced 2- to 8-fold. Positive controls produced strong ISG expression as expected. The responses were not strictly dose-dependent within the tested range (0.5–1 μg). These results demonstrated that the 3′UTR pseudoknot structures of Arteriviruses function as potent PAMPs capable of activating type I IFN and ISG responses.

### 3.3. Effects of Arterivirus Pseudoknot RNAs on the mRNA Expression of RNA-Related Pattern Recognition Receptors

Marc145 cells were transfected with increasing doses (0.5, 0.75, or 1 μg) of the 3′UTR pseudoknot RNAs from PRRSV-BJ4, SHFV, EAV, and LDV. At 12 h post-transfection, the mRNA expression levels of several RNA-associated PRRs (RIG-I, MDA5, TLR3, TLR7, TLR8, and TLR9) were measured by qRT-PCR. Poly(I:C) and 3RP were used as positive controls, and all values were normalized to mock-transfected controls.

As shown in Figure 3A–F, all four pseudoknots markedly upregulated RIG-I and TLR8 expression across multiple concentrations. For PRRSV-BJ4, RIG-I was induced 5.7-fold, while TLR8 was induced up to 22.3-fold (Figure 3A–F). The SHFV pseudoknot elicited a particularly strong RIG-I response (up to 18.4-fold) and also induced TLR8 (up to 38.8-fold) (Figure 3A–F). EAV pseudoknot induced a very strong RIG-I response (up to 21-fold) and a robust TLR8 induction (up to 35-fold) (Figure 3A–F). LDV pseudoknot increased RIG-I (up to 8.7-fold) and TLR8 (up to 21.5-fold), with modest TLR7 upregulation (Figure 3A–F). In contrast, MDA5, TLR3, and TLR9 were weakly or variably induced, and TLR7 showed no consistent changes (Figure 3A–F). MDA5, TLR3, and TLR9 were analyzed using PRRSV-BJ4 and SHFV as representative pseudoknots. Positive controls 3RP and poly(I:C) induced broad PRR upregulation as expected, including strong RIG-I and TLR8 responses. These results identified RIG-I and TLR8 as the primary PRRs consistently activated by Arterivirus pseudoknot RNAs.

### 3.4. Key Signaling Nodes in the Type I IFN Response Induced by Arterivirus 3′ UTR Pseudoknots

Marc145 cells were transfected with increasing doses (0.5, 0.75, or 1 μg) of the 3′UTR pseudoknot RNAs from PRRSV-BJ4, SHFV, EAV, and LDV. At 12 h post-transfection, the mRNA expression levels of type I IFN pathway-related molecules (MyD88, MAPK1, MAPK8, NEMO, DDX3X, IKKα, IKKβ, MAVS) and downstream targets (p50, p65, AP-1, TBK1, IKKε, IRF3, IRF7) were measured by qRT-PCR. Poly(I:C) and 3RP were used as positive controls, and all values were normalized to mock-transfected controls.

As shown in Figure 4, PRRSV-BJ4, SHFV, and LDV pseudoknots significantly upregulated the key adaptor molecule MyD88 (up to 7.9-fold for PRRSV-BJ4, 5.8-fold for SHFV, 7-fold for EAV, and 8-fold for LDV) and IRF7 (up to 42-fold for PRRSV-BJ4, 26-fold for SHFV, and 49.7-fold for LDV), whereas the EAV pseudoknot for IRF7 showed a comparable but non-significant trend at the tested doses (Figure 4A,O). AP-1 was also markedly induced by PRRSV-BJ4 and SHFV (up to 8.8-fold), and to a lesser extent by EAV and LDV. In contrast, TBK1 and IKKε showed little to no induction (mostly ≤ 2-fold), and IRF3 was not uniformly induced by all pseudoknots. However, SHFV pseudoknot at 0.75 and 1 μg significantly upregulated IRF3 expression (Figure 4N). Among the upstream adaptors, MAVS was modestly induced (≤2.6-fold) by all pseudoknots, while MAPK1, MAPK8, NEMO, DDX3X, IKKα, and IKKβ were moderately elevated (generally 1.5–3-fold) for PRRSV-BJ4 and SHFV. PRRSV-BJ4 and SHFV were used as representative pseudoknots for downstream signaling screening; EAV and LDV were not included in this panel. p50 and p65 were also increased (up to 5.8-fold for SHFV and 1.8-fold for PRRSV-BJ4, respectively) (Figure 4I,J). Positive controls 3RP and poly(I:C) induced broad activation of the pathway, including strong upregulation of MyD88, AP-1, IRF7, and p50. These results indicated that the Arterivirus pseudoknots activate type I IFN signaling primarily through the MyD88-IRF7 axis, with variable engagement of other components.

### 3.5. Key Signaling Nodes in the ISG Response Induced by Arterivirus 3′UTR Pseudoknots

Marc145 cells were transfected with increasing doses (0.5, 0.75, or 1 μg) of the 3′ UTR pseudoknot RNAs from PRRSV-BJ4, SHFV, EAV, and LDV. At 12 h post-transfection, the mRNA expression levels of JAK-STAT pathway-related molecules (TYK2, JAK1, STAT1, STAT2, STAT3, STAT4, STAT5, STAT6, IRF9, MX1, OAS1a) were measured by qRT-PCR. Poly(I:C) and 3RP were used as positive controls, and all values were normalized to mock-transfected controls.

As shown in Figure 5A–K, all four pseudoknots consistently and markedly upregulated STAT2 and IRF9 across multiple concentrations. For PRRSV-BJ4, STAT2 was induced up to 19-fold, and IRF9 up to 3.9-fold; STAT1, STAT3, JAK1, and OAS1a were modestly increased (approximately 1.5–2.5-fold), while TYK2 showed no significant change, and STAT4, STAT5, and MX1 remained below mock levels. For SHFV, STAT2 was induced up to 9.1-fold, IRF9 up to 13.5-fold, and MX1 and OAS1a were also increased (up to 3.4-fold and 3.8-fold, respectively); STAT4, STAT5, and STAT6 were below mock levels. STAT3, STAT4, STAT5, STAT6, MX1, and OAS1a were analyzed using PRRSV-BJ4 and SHFV as representative pseudoknots. For EAV, STAT2 was induced up to 12-fold, IRF9 up to 5.7-fold, and JAK1 and STAT1 showed modest increases; STAT4 remained below mock, and data for several other targets were not obtained. For LDV, STAT2 was induced up to 15.4-fold, IRF9 up to 4.3-fold, and STAT1 was modestly increased; STAT4 remained below mock. Positive controls 3RP and poly(I:C) induced strong activation of STAT2 (up to 24-fold and 38.7-fold, respectively) and IRF9 (up to 11-fold and 12.7-fold). These results indicated that the Arterivirus pseudoknots activate the JAK-STAT pathway primarily through upregulation of STAT2 and IRF9, which are key components of the ISGF3 complex driving ISG transcription.

### 3.6. The Arterivirus 3′ UTR Pseudoknot RNA Acts as a PAMP to Induce Type I IFN via RIG-I and TLR8

To determine whether RIG-I and TLR8 are essential for the pseudoknot-induced innate immune response, shRNA-mediated knockdown experiments were performed. Marc145 cells were transfected with shRNA plasmids targeting RIG-I or TLR8 for 48 h, followed by transfection with the optimal concentration (determined in preliminary experiments) of the 3′ UTR pseudoknot RNAs from PRRSV, LDV, EAV, and SHFV. At 12 h post-transfection, the mRNA expression levels of RIG-I, TLR8, IFNβ, PKR, and ISG56 were measured by qRT-PCR. As shown in Figure 6, knockdown of RIG-I or TLR8 significantly reduced the pseudoknot-induced expression of all five genes for each of the four Arteriviruses.

### 3.7. Structural Analysis of the pRIG-I-RD and PRRSV 3′ UTR Pseudoknot RNA Complex by Small-Angle X-Ray Scattering (SAXS)

The in vitro transcribed PRRSV 3′ UTR pseudoknot RNA and high-purity pRIG-I-RD protein were mixed at a molar ratio of 1:2 and incubated on ice for 1 h to form the complex. The complex was then purified by gel filtration chromatography on a Superdex 200 10/300 GL column. SAXS was employed to study the solution structure of the pRIG-I-RD/PRRSV 3′ UTR pseudoknot RNA complex. SAXS data were collected and subjected to structural analysis (Figure 7A). Guinier analysis yielded an Rg value of 36.19 Å, an sRg range of 0.73–1.3, and a zero-angle scattering intensity Io of 18.11. The sRg value of the pRIG-I-RD-RNA complex was less than 1.3, within a reasonable range. Most data points fell on a straight line with minimal fluctuation in the bottom error bars, indicating high-quality SAXS data suitable for further analysis (Figure 7C). GNOM analysis of the pRIG-I-RD-RNA complex showed good agreement between the experimental curve and the theoretical curve at a Dmax value of 161.47 Å. The P(*r*) function exhibited a gentle and uniform tail at the junction with the abscissa in the large r region, and the P(*r*) curve showed two peaks, suggesting that the pRIG-I-RD-RNA complex was an elongated macromolecular structure possibly containing multiple domains with at least two centers (Figure 7B,D). Data processing and structural analysis were performed using Primus software and the Chimera program, revealing the basic shape of the complex in solution. Superimposition of the experimental envelope (shown as surface) onto the model of the pRIG-I-RD/PRRSV 3′ UTR pseudoknot RNA complex is represented in cartoon format (Figure 7E). This analysis provides structural evidence for RIG-I recognition of the pseudoknot RNA, consistent with the established role of the RIG-I RD in sensing higher-order RNA structures. A direct structural analysis of TLR8–pseudoknot interaction was not performed in the present study and remains to be addressed in future work, given that TLR8 recognizes RNA through an endosomal, proteolytically processed, and dimerization-dependent mechanism distinct from that of RIG-I.

### 3.8. Proposed Model for Pseudoknot-Induced Activation of Type I IFN and ISG Responses

The present study demonstrates that conserved 3′ UTR pseudoknot structures exist in the Arteriviruses EAV, PRRSV, LDV and SHFV. These pseudoknots act as PAMPs and are recognized by the host PRRs RIG-I and TLR8. They induce type I IFN responses by targeting both the RIG-I and TLR8 signaling pathways, and subsequently activate ISG responses (Figure 8).

## 4. Discussion

During infection with RNA viruses, the host recognizes pathogen-associated molecular patterns (PAMPs) derived from viral RNAs through pattern recognition receptors (PRRs), thereby initiating innate immune responses. As a key product of the innate immune response, IFN-I is critical for antiviral defense. This study investigated the role of the 3′ UTR pseudoknot structures of Arteriviridae members in activating type I IFN responses and explored the underlying molecular mechanisms. The results demonstrate that these conserved RNA structures act as PAMPs recognized by host PRRs, thereby inducing potent IFN-I and ISG responses. These findings provide new insights into understanding immune activation during Arterivirus infection.

Bioinformatics analysis confirmed that the 3′ UTRs of EAV, LDV, SHFV and PRRSV contain evolutionarily conserved pseudoknot structures (Figure 1), suggesting that these structures serve critical biological functions rather than being randomly formed. The present study showed that transfection with each pseudoknot RNA significantly upregulated the mRNA expression of IFNα, IFNβ, and multiple ISGs (including PKR, ISG15, and ISG56) in Marc145 cells (Figure 2). The magnitude of induction was not strictly dose-dependent within the tested range (0.5–1 μg). These findings confirm that the 3′ UTR pseudoknot-containing region represents a novel class of PAMP capable of effectively activating type I IFN responses. It should be noted that structure-disrupting mutants were not used in this study; therefore, a contribution from other sequence or structural elements within this RNA fragment cannot be fully excluded, and the precise contribution of the pseudoknot fold itself remains to be defined. Notably, the magnitude of ISG induction varied among the four pseudoknots: EAV and LDV induced extremely high ISG56 expression, whereas PRRSV and SHFV induced more moderate levels (Figure 2C–E). The magnitude of ISG induction varied among the four pseudoknots. The biological significance of these differences remains unclear and requires further investigation.

Viral RNA triggers the IFN response through engagement with RNA-associated PRRs. To identify the host sensors involved, the expression of several RNA-associated PRRs was examined. All four pseudoknots consistently upregulated RIG-I and TLR8 expression (Figure 3A–F). shRNA-mediated knockdown experiments further demonstrated that silencing either RIG-I or TLR8 significantly reduced the pseudoknot-induced expression of IFNβ, PKR, and ISG56 (Figure 6), which suggested that RIG-I and TLR8 are its host sensors. Previous studies have shown that double-stranded RNA with 5′-triphosphate groups [16], long double-stranded RNA with 5′-diphosphate groups [17], blunt-ended stem-loop structures formed during replication of some negative-sense RNA viruses [18], and “abortive RNA” produced during error-prone viral replication [19] serve as a potent agonist of RIG-I. The conformationally changed mRNA of paramyxoviruses [20] and the hairpin RNA of influenza virus [21] can bind and activate MDA5. AU-rich RNA fragments [22] can simultaneously activate both RIG-I and MDA5. A recent study indicated that dsRNAs beginning with 5′-pppA trigger a stronger RIG-I/IFN response than those beginning with 5′-pppG [23]. These results also show that the RNAs with pseudoknots of Arterivirus represent a class of PAMPs. This study showed that TLR8 is involved in Arterivirus RNA sensing. It should be noted that direct binding of TLR8 to the pseudoknot RNA was not examined in this study, and direct recognition by TLR8 remains to be verified in future work. While previous studies have identified various viral RNA structures (e.g., 5′-triphosphate dsRNA, AU-rich sequences) as ligands for RIG-I or TLR7/8 [16,22,24], the function of the Arterivirus pseudoknot was previously unclear.

Our data indicated that Arterivirus 3′ UTR pseudoknots predominantly activate the MyD88–IRF7 signaling axis, whereas MAVS was modestly activated (Figure 4A,H,O). In addition, the pseudoknots significantly upregulated STAT2 and IRF9 (Figure 5), suggesting that type I IFN signaling may be further amplified through the JAK–STAT–IRF9 axis. This suggests the existence of receptor cooperation and signaling crosstalk during Arterivirus recognition. Notably, the regulation of porcine TLR8 signaling has been shown to be finely tuned by immune checkpoint molecules. T-cell immunoglobulin and mucin domain-containing protein 3 (TIM-3) negatively regulates porcine Toll-like receptor 8 (pTLR8) signaling by inhibiting the phosphoinositide 3 kinase-AKT (PI3K-AKT) pathway, thereby disturbing pTLR8-mediated antiviral activity [25]. These observations highlight the complexity of TLR8-mediated immune regulation in swine, which may influence the outcome of Arterivirus infection in natural hosts.

Pathway analysis revealed that downstream signaling molecules, including MyD88, IRF7, and STAT2, were all activated by the pseudoknots (Figure 4 and Figure 5). These data suggest that the pseudoknot engages both the TLR8-MyD88-IRF7 and RIG-I-IRF7 signaling axes, which ultimately converge on the formation of the ISGF3 complex (STAT1-STAT2-IRF9) to drive ISG transcription (Figure 5). This finding aligns with the established role of MAVS in antiviral innate immunity [26,27]. This study validates the pseudoknot as an upstream trigger in Arteriviruses. The SAXS results are consistent with the formation of a complex between the pseudoknot RNA and RIG-I, which may help explain its immunostimulatory activity. As SAXS provides low-resolution overall shape information in solution, it cannot define the specific binding interface or key residues; high-resolution structural methods such as crystallography or cryo-electron microscopy (cryo-EM) will be required to resolve these details. The results of SAXS provide structural evidence that RIG-I can recognize the pseudoknot structure. It should be noted that the present study provides functional, rather than direct structural, evidence for TLR8 involvement in pseudoknot sensing. Although TLR8 was consistently induced by the pseudoknot RNAs and its knockdown significantly reduced the type I IFN response, a direct TLR8–pseudoknot interaction remains to be demonstrated. TLR8 recognizes RNA within endosomes and requires proteolytic processing and dimerization for activation, which differs fundamentally from the cytoplasmic RD-mediated recognition of RNA by RIG-I. Future studies using endosome-based or cell-based TLR8 activation systems will be needed to define whether and how TLR8 physically engages the pseudoknot RNA. These findings expand the known repertoire of RIG-I ligands by demonstrating that complex higher-order RNA structures can serve as high-affinity ligands for RIG-I, broadening the structural basis of RIG-I ligand recognition [28]. Recent high-resolution crystal structures of duck RIG-I bound to non-canonical duplexes mimicking the influenza virus viral RNA promoter (panhandle) have further elucidated the molecular details of RIG-I recognition of complex RNA structures (PDB: 9TM6). These structural insights, together with the present SAXS analysis, reinforce the concept that RIG-I can recognize a diverse range of higher-order RNA architectures beyond canonical double-stranded RNA ligands. Moreover, earlier structural studies revealed that the C-terminal regulatory domain (RD) of RIG-I functions as the primary RNA 5′-triphosphate sensor, and that RNA binding induces conformational changes that relieve autoinhibition and allow caspase activation and recruitment domain (CARD) domain activation [29]. These mechanistic insights support the interpretation that the PRRSV pseudoknot, as a complex higher-order RNA structure, is likely recognized by the RIG-I RD in a manner analogous to other non-canonical RNA ligands.

Viral PAMPs have significant implications for antiviral therapy and vaccine development. The 5′ replication-defective interfering genomic RNA in the live attenuated rabies virus vaccine acts as an endogenous adjuvant that significantly stimulates IFN responses by enhancing RIG-I [30]. The RIG-I-like receptor (RLR) agonist M8, an optimized vesicular stomatitis virus (VSV)-UTR 5′pppRNA, protects mice against influenza A virus (IAV) H5N1 recombinant (H5N1-RE) and chikungunya virus (CHIKV) infection [31,32]. The 3′ UTR pseudoknot-containing region represents a novel class of PAMPs capable of effectively activating type I IFN responses and provides ideal candidate molecules for the development of immune adjuvants or RNA vaccines against Arterivirus infections. Furthermore, the important role of the TLR8 pathway in recognizing viral RNA identified here offers a new drug target for swine disease control. Small-molecule TLR8 agonists or antagonists could be used to modulate host immune status by enhancing innate immunity. The potential of TLR8 agonists as antiviral agents has been further supported by recent studies demonstrating that TLR8 activation, in combination with other innate immune agonists, can effectively reverse viral latency and enhance antiviral immunity [33]. This suggests that TLR8-targeting strategies may have broader applications in viral disease control.

Recent advances in Arterivirus biology have also revealed novel aspects of viral entry and immune evasion. For example, CD81 has been identified as an alternative receptor for equine arteritis virus (EAV), demonstrating that Arteriviruses can switch receptor usage to expand their cellular tropism [34]. Additionally, computational modeling of the EAV GP5/M dimer has provided molecular insights into immune evasion mechanisms involving antigenic drift and glycan shielding [35]. These findings collectively highlight the complex interplay between Arteriviruses and the host immune system, underscoring the need for continued investigation into viral PAMP recognition and immune modulation. Furthermore, a comprehensive study of innate immune antagonism and immune evasion strategies of PRRSV has recently highlighted how multiple viral proteins actively suppress antiviral responses, allowing PRRSV to persist and spread [36,37,38,39,40], which is also observed in other Arteriviruses [41,42,43,44]. These observations underscore the importance of identifying and characterizing viral PAMPs such as the 3′ UTR pseudoknot, as they may represent vulnerable targets for therapeutic intervention.

Several limitations of this study should be acknowledged. First, all experiments were performed in Marc145 cells (African green monkey kidney epithelial cells). Although this is a classical in vitro system for Arterivirus (especially PRRSV) research, its non-natural host origin and non-professional immune cell type may limit the generalizability of the conclusions. TLR8 expression and function differ among species, and species-specific differences in TLR8 RNA recognition may influence the interpretation of our results. It remains unclear whether Marc145 cells fully recapitulate the immune status of natural target cells such as PAMs, and future validation in porcine primary cells will be important. Moreover, all transfections used in vitro transcribed RNA to mimic viral infection, which, while facilitating controlled variable analysis and focusing on structure–function relationships, does not fully reproduce the spatiotemporal distribution, copy number, or concurrent viral protein regulatory effects during natural infection. Future studies should extend to more physiologically relevant models such as primary PAMs and validate findings using infectious clones or viral particles. Second, while the SAXS analysis provided a low-resolution envelope of the complex, high-resolution structural data obtained by X-ray crystallography or cryo-EM are needed to precisely define the interaction interface, key nucleotides, and protein residues. Additionally, the mechanism by which the pseudoknot activates TLR8 remains unclear. A direct TLR8–pseudoknot interaction analysis was not performed in this study. While the functional knockdown data strongly support a role for TLR8, the structural basis of TLR8 recognition of the pseudoknot RNA remains to be established. Given that TLR8 activation depends on endosomal processing and dimerization, future studies should employ endosome-based or cell-based systems rather than direct biophysical binding assays to interrogate this interaction. Future studies should aim for co-crystallization or cryo-EM structural determination and employ fluorescence reporter systems to monitor TLR8 activation dynamics in endosomes. Third, although the results demonstrate immune activation at the mRNA levels, the secretion of functional IFN protein or the antiviral state was not verified. Whether the induced response produces biologically active IFN-I and effectively inhibits live virus replication remains to be tested. Subsequent studies should measure IFN-β protein secretion by enzyme-linked immunosorbent assay (ELISA) and assess viral replication titers after live virus challenge to confirm functional consequences. The downstream signaling panel (MAPK1, MAPK8, NEMO, DDX3X, IKKα, IKKβ, p65, and IKKε) and JAK-STAT panel (STAT3, STAT4, STAT5, STAT6, MX1, and OAS1a) was examined using PRRSV-BJ4 and SHFV as representative pseudoknots. A complete comparative analysis across all four pseudoknots will be valuable in future work. Fourth, a parallel negative control such as shRNA-TLR7 was not included in the present knockdown experiments. Although TLR7 was not consistently induced by the pseudoknot RNAs in our PRR screening, we acknowledge that an shRNA-TLR7 control would further strengthen the specificity of our conclusion. Future studies will be needed to fully address this point.

In summary, this study makes several important contributions. First, it reveals the immune-activating function of a conserved RNA element in Arteriviruses at the molecular level. Second, it identifies TLR8 as a novel receptor for viral RNA recognition, offering a potential target for antiviral drug or vaccine adjuvant development. Third, the differential immunostimulatory potency among the four pseudoknots may partially explain the varying pathogenicity of different Arteriviruses. Finally, the findings suggest that viruses may utilize such structures to moderately activate immune responses during early infection to modulate the replication environment, and whether such immunomodulation contributes to viral pathogenicity warrants further investigation. This study provides important scientific bases and translational targets for antiviral immune therapy development, vaccine design, and genetic disease resistance breeding.

## Figures and Tables

**Figure 1:**
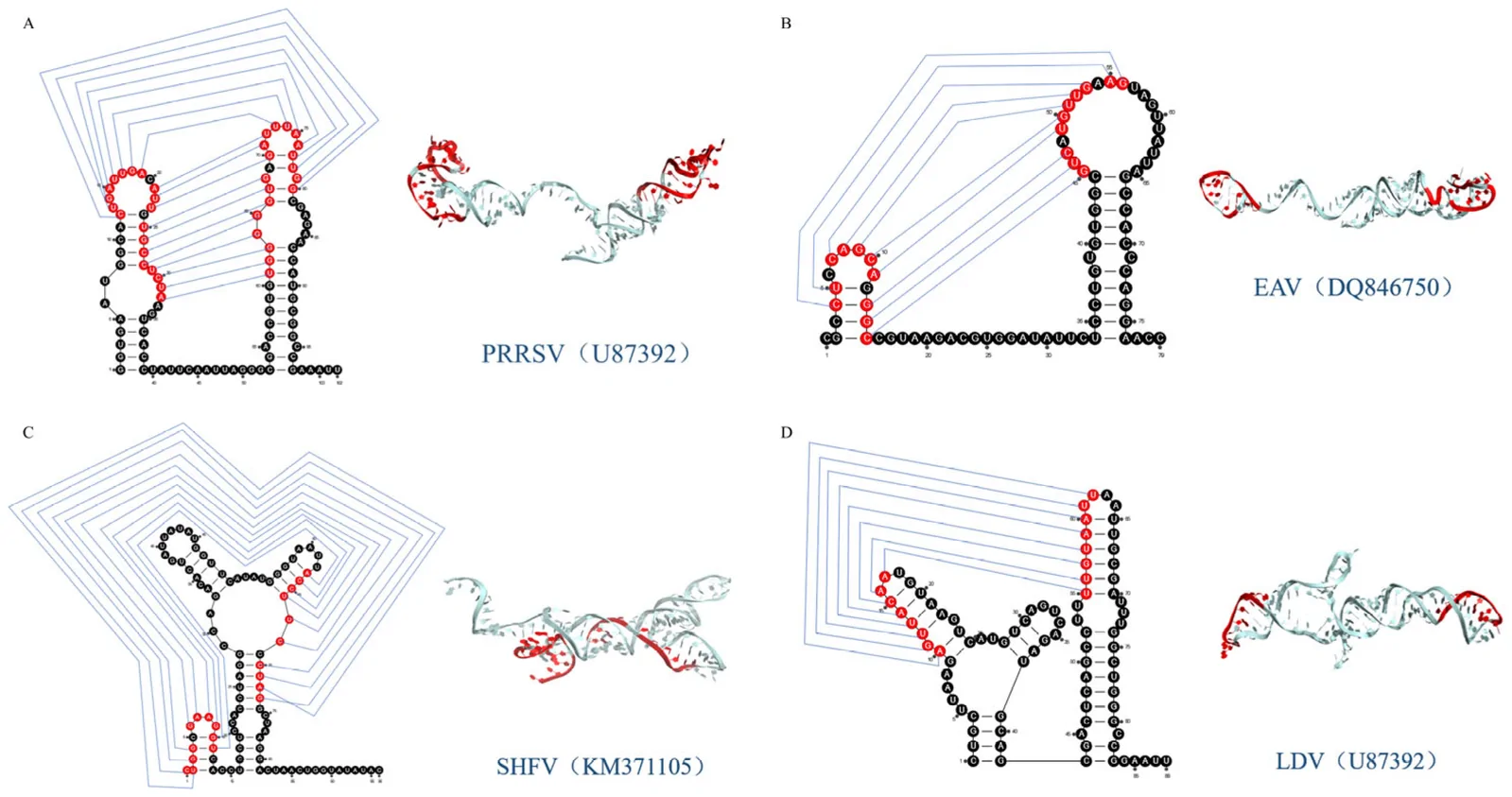
Structural prediction of the 3′ UTR pseudoknot in Arteriviridae members. Predicted secondary structures and tertiary conformations of the 3′ UTR pseudoknot RNAs from PRRSV-BJ4 (**A**), EAV (**B**), SHFV (**C**) and LDV (**D**). Interactions between loop structures are indicated by grey lines. Structures were predicted using the bioinformatics software RNAalifold (ViennaRNA Package v2.6.4), RNArtist (version 1.1.3), and RNAComposer (https://rnacomposer.cs.put.poznan.pl/, accessed on 5 May 2024).

**Figure 2:**
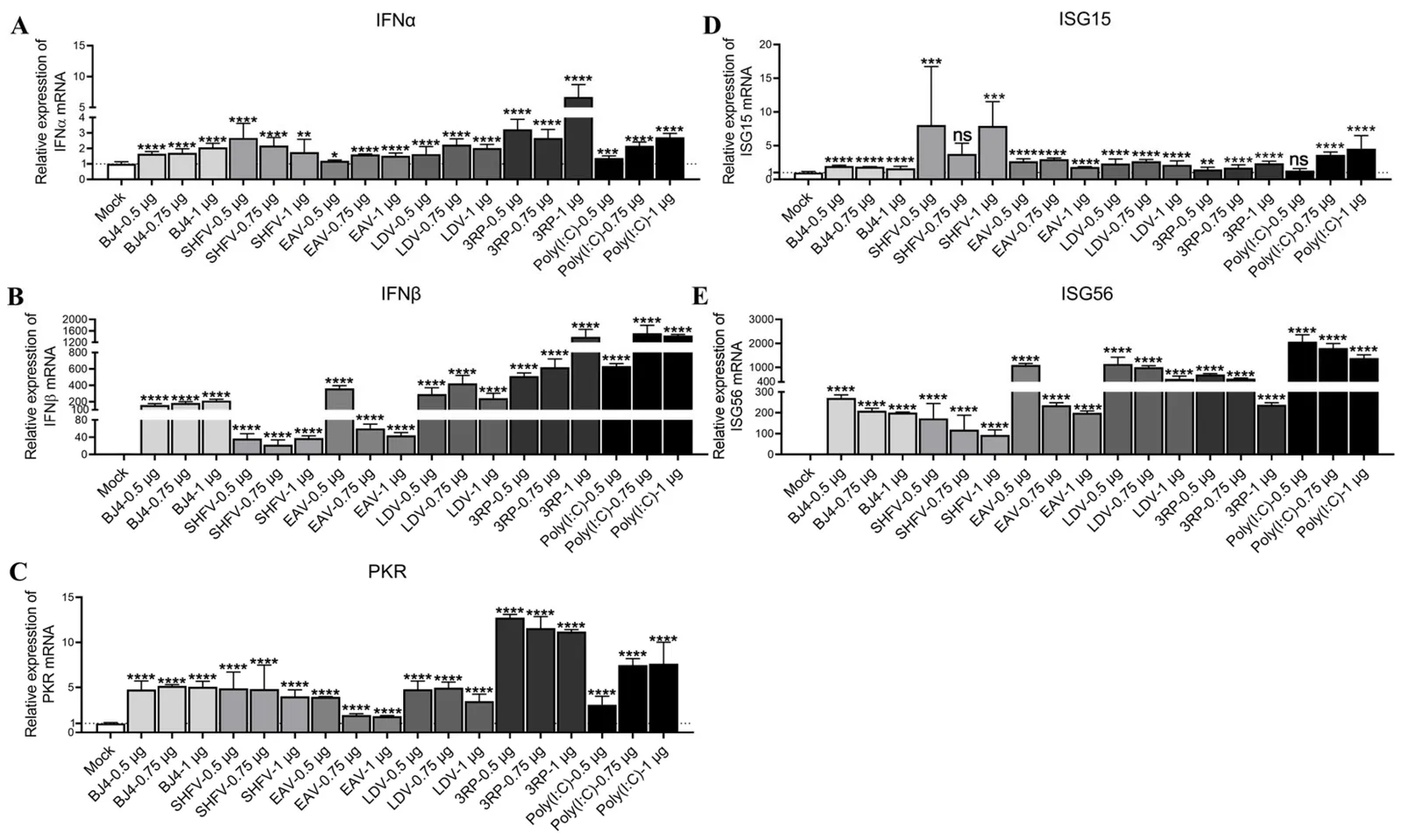
Upregulation of type I IFN and ISG mRNA expression by Arterivirus pseudoknot RNAs. Marc145 cells were transfected with 0.5, 0.75, or 1 μg of in vitro transcribed 3′ UTR pseudoknot RNAs from PRRSV-BJ4, SHFV, EAV, and LDV. At 12 h post-transfection, mRNA levels were measured by qRT-PCR. (**A**,**B**) IFNα and IFNβ expression. (**C**–**E**) PKR, ISG15 and ISG56 expression. Poly(I:C) and 3RP were used as positive controls. All values are fold changes relative to mock-transfected control. Detailed fold changes are provided in the Section 3. Statistical comparisons: BJ4, SHFV, EAV, and LDV vs. Mock (* *p* < 0.05, ** *p* < 0.01, *** *p* < 0.001, **** *p* < 0.0001, ns = not significant); data are presented as mean ± SD.

**Figure 3:**
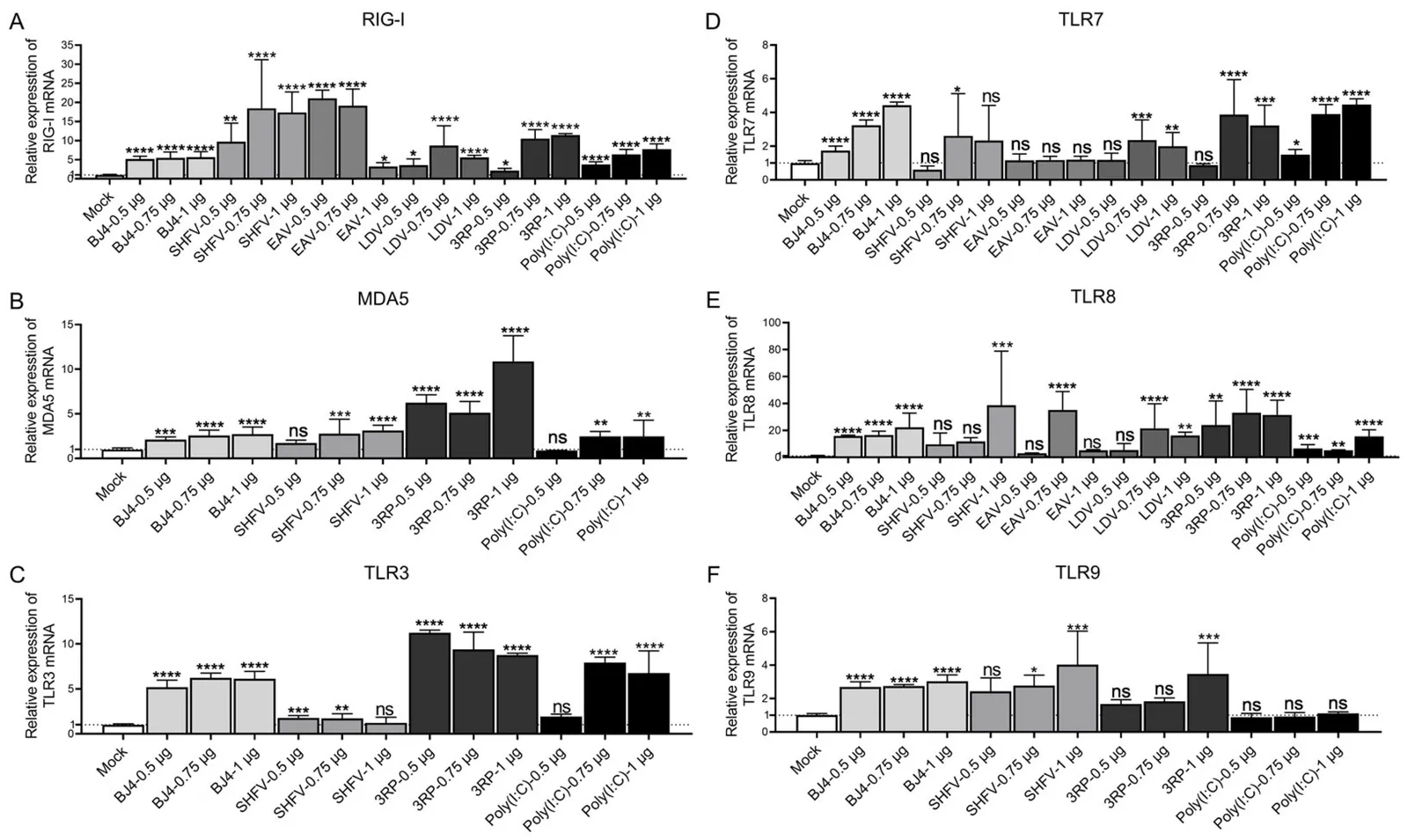
Effects of Arterivirus pseudoknot RNAs on the mRNA expression of RNA-related pattern recognition receptors. (**A**–**F**) Marc145 cells were transfected as in Figure 2. mRNA levels of RIG-I, MDA5, TLR3, TLR7, TLR8 and TLR9 in cells stimulated with PRRSV, SHFV, EAV, and LDV pseudoknot RNAs were measured by qRT-PCR. Positive controls: 3RP and poly(I:C). Data are fold changes relative to mock control. Statistical comparisons: BJ4, SHFV, EAV, and LDV vs. Mock (* *p* < 0.05, ** *p* < 0.01, *** *p* < 0.001, **** *p* < 0.0001, ns = not significant); data are presented as mean ± SD.

**Figure 4:**
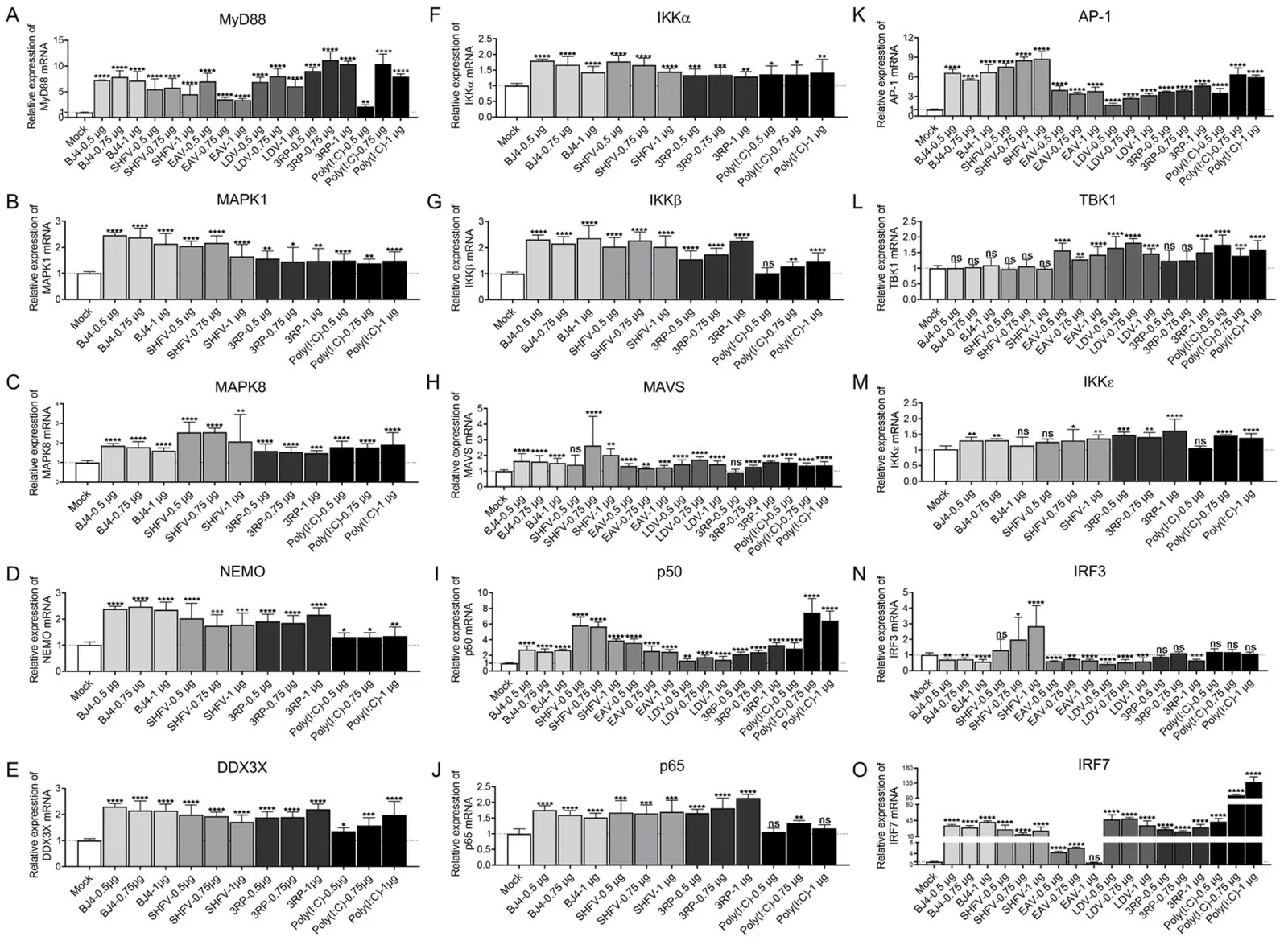
Key signaling nodes in the type I IFN response induced by Arterivirus 3′ UTR pseudoknots. (**A**–**O**) Marc145 cells were transfected as in Figure 2. Expression of MyD88, MAPK1, MAPK8, NEMO, DDX3X, IKKα, IKKβ, MAVS, p50, p65, AP-1, TBK1, IKKε, IRF3, and IRF7 in cells stimulated with PRRSV-BJ4, SHFV, EAV, and LDV pseudoknot RNAs. MAPK1, MAPK8, NEMO, DDX3X, IKKα, IKKβ, p65, and IKKε were analyzed using PRRSV-BJ4 and SHFV as representative pseudoknots. Data are shown for PRRSV-BJ4, SHFV, EAV and LDV. Positive controls: 3RP and poly(I:C). All values are fold changes relative to mock control. Statistical comparisons: BJ4, SHFV, EAV, and LDV vs. Mock (* *p* < 0.05, ** *p* < 0.01, *** *p* < 0.001, **** *p* < 0.0001, ns = not significant); data are presented as mean ± SD.

**Figure 5:**
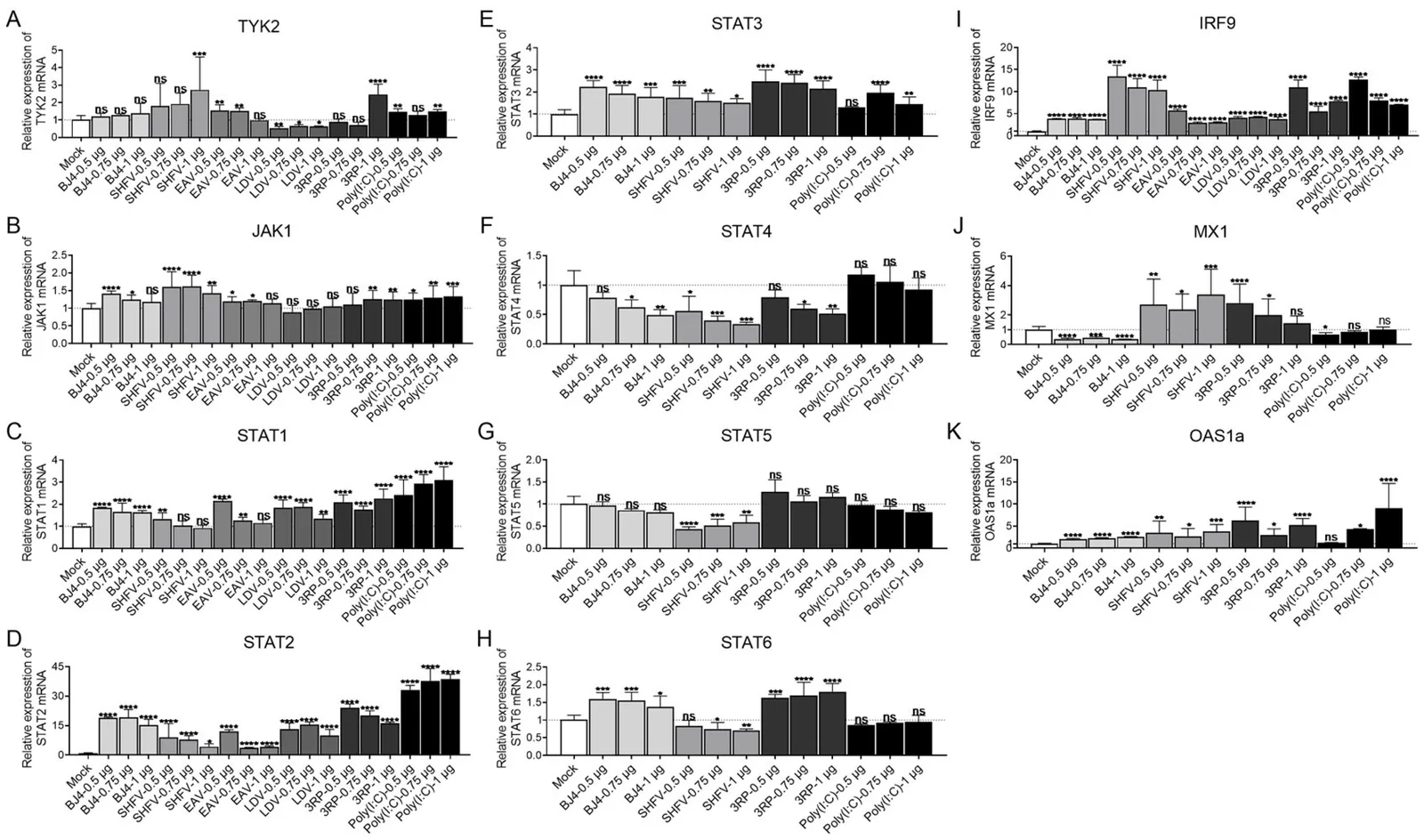
Key signaling nodes in the ISG response induced by Arterivirus 3′ UTR pseudoknots. (**A**–**K**) Marc145 cells were transfected as in Figure 2. Expression of TYK2, JAK1, STAT1, STAT2, STAT3, STAT4, STAT5, STAT6, IRF9, MX1, and OAS1a in cells stimulated with PRRSV-BJ4, SHFV, EAV, and LDV pseudoknot RNAs. STAT3, STAT4, STAT5, STAT6, MX1, and OAS1a were analyzed using PRRSV-BJ4 and SHFV as representative pseudoknots. Positive controls: 3RP and poly(I:C). All values are fold changes relative to mock control. Statistical comparisons: BJ4, SHFV, EAV, and LDV vs. Mock (* *p* < 0.05, ** *p* < 0.01, *** *p* < 0.001, **** *p* < 0.0001, ns = not significant); data are presented as mean ± SD.

**Figure 6:**
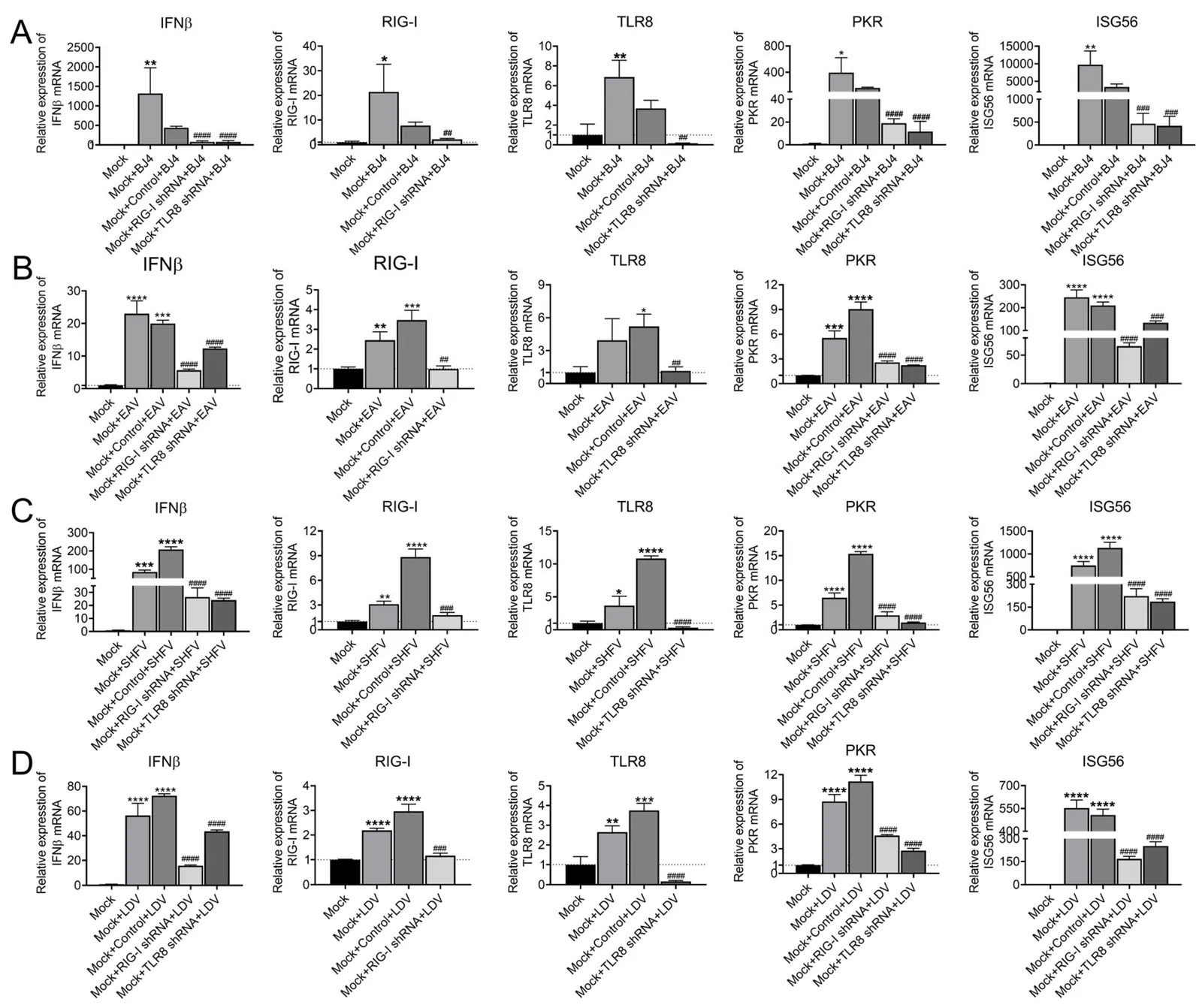
The Arterivirus 3′ UTR pseudoknot RNA acts as a PAMP to induce type I IFN via RIG-I and TLR8. (**A**–**D**) Marc145 cells were transfected with shRNA plasmids targeting RIG-I or TLR8 for 48 h, followed by transfection with the optimal concentration of the 3′ UTR pseudoknot RNAs from PRRSV, LDV, EAV, and SHFV. At 12 h post-transfection, mRNA levels of RIG-I, TLR8, IFNβ, PKR and ISG56 were measured by qRT-PCR. Statistical comparisons: Mock + BJ4 and Mock + Control + BJ4 vs. Mock alone (* *p* < 0.05, ** *p* < 0.01, *** *p* < 0.001, **** *p* < 0.0001); Mock + RIG-I shRNA + BJ4 and Mock + TLR8 shRNA + BJ4 vs. Mock + Control + BJ4 (^##^ *p* < 0.01, ^###^ *p* < 0.001, ^####^ *p* < 0.0001). Data are presented as mean ± SD.

**Figure 7:**
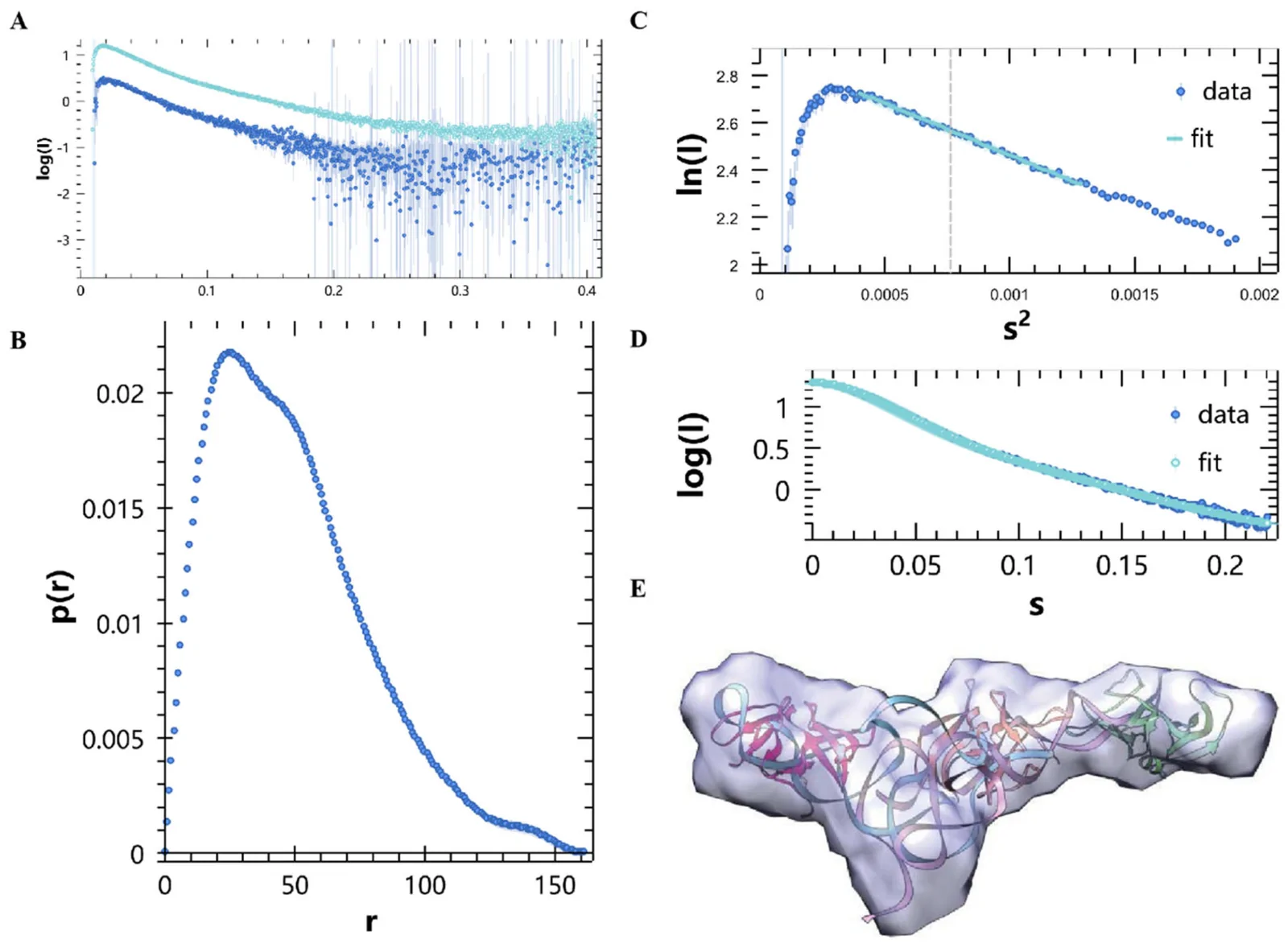
Structural analysis of the pRIG-I-RD and PRRSV 3′ UTR pseudoknot RNA complex by small-angle X-ray scattering (SAXS). In vitro transcribed PRRSV 3′ UTR pseudoknot RNA and high-purity pRIG-I-RD protein were mixed at a molar ratio of 1:2 and incubated on ice for 1 h to form the complex, which was purified by gel filtration. (**A**) SAXS data collection and structural analysis scheme. (**B**,**D**) GNOM analysis: Dmax = 161.47 Å, good agreement between experimental and theoretical curves; the P(*r*) function shows two peaks, suggesting an elongated macromolecular structure with multiple domains. (**C**) Guinier analysis: Rg = 36.19 Å, sRg range 0.73–1.3, Io = 18.11, indicating high-quality data. (**E**) Superimposition of the experimental envelope (surface) onto the model of the pRIG-I-RD/PRRSV 3′ UTR pseudoknot RNA complex (cartoon). Data processing used primus/qt ATSAS 3.3.0 (r14945) and chimera-1.12.

**Figure 8:**
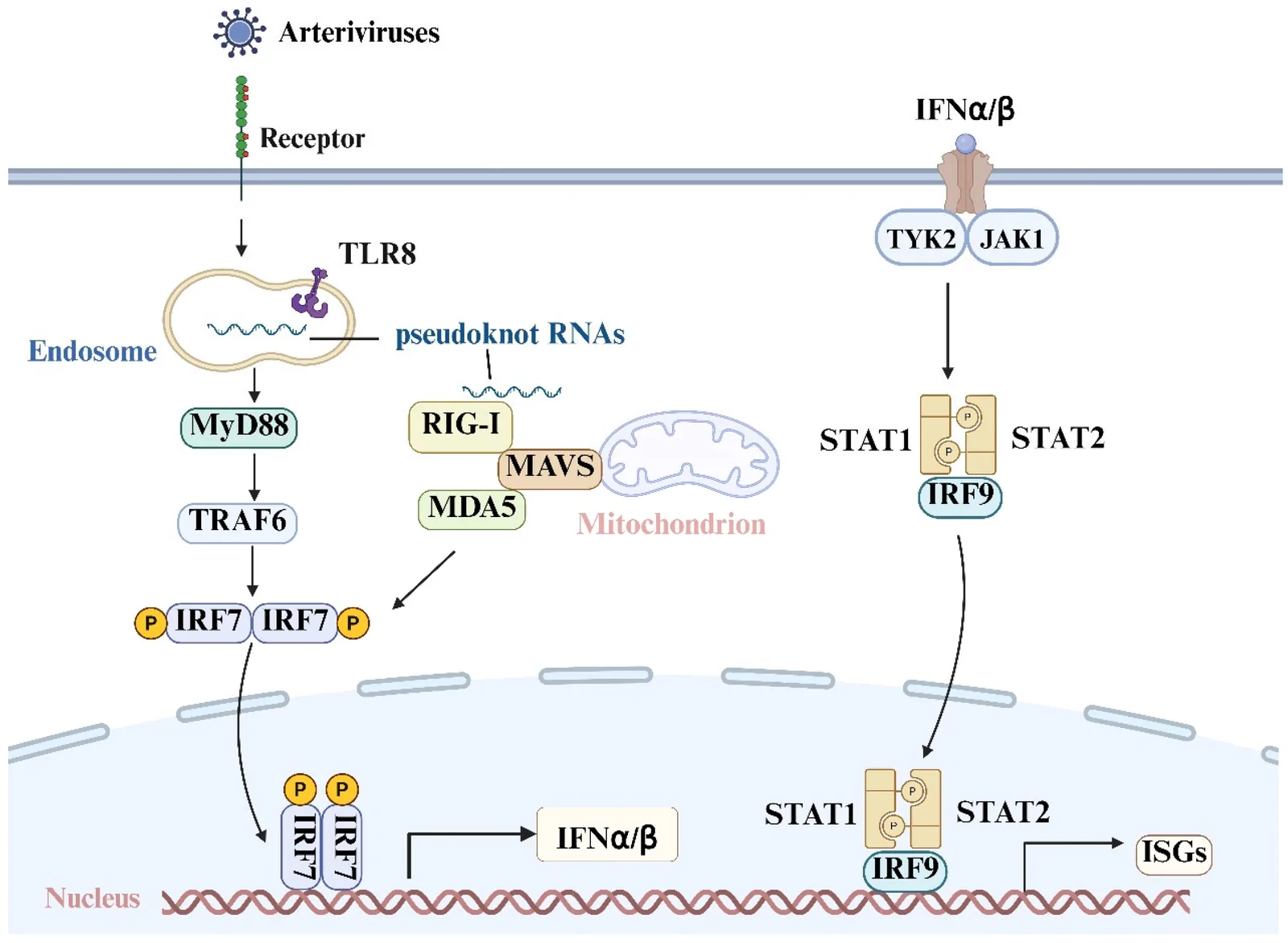
Proposed model for pseudoknot-induced activation of type I IFN and ISG responses. The figure was generated using BioRender, an online tool available at https://app.biorender.com/. (Copyright Code: PB29NGTR7Z).

**Table 1:** Specific DNA sequences containing T7 promoters.

Primer Name	Sequence
PRRSV pseudoknot 50–151 F	TAATACGACTCACTATAGGGGGTGAATGGCACTGATTGACATTGTGCCTCTAAGTCACCTATTCAATTAGGGCGACCGTGTGGGGGTGAGATTTAATTGGCGAGAACCATGCGGCCGAAATT
PRRSV pseudoknot 50–151 R	/i2OMeA//i2OMeA/TTTCGGCCGCATGGTTCTCGCCAATTAAATCTCACCCCCACACGGTCGCCCTAATTGAATAGGTGACTTAGAGGCACAATGTCAATCAGTGCCATTCACCCCCTATAGTGAGTCGTATTA
SHFV pseudoknot F	TAATACGACTCACTATAGGGCTGGCGAAGGTCACCTCCTCCACCTAGGCCAGACACTGATTATATGGTTCATATGGGTAATTACCTTCCCTAGGCTAAGGACTAACTGGTATATAC
SHFV pseudoknot R	/i2OMeG//i2OMeU/ATATACCAGTTAGTCCTTAGCCTAGGGAAGGTAATTACCCATATGAACCATATAATCAGTGTCTGGCCTAGGTGGAGGAGGTGACCTTCGCCAGCCCTATAGTGAGTCGTATTA
EAV pseudoknot F	TAATACGACTCACTATAGGGCGCCTCCAGCAGGGCCGTAAGACGTGGATATTCTCCTGTGTGGCGTCATGTTGAAGTAGTTATTAGCCACCCAGGAACC
EAV pseudoknot R	/i2OMeG//i2OMeG/TTCCTGGGTGGCTAATAACTACTTCAACATGACGCCACACAGGAGAATATCCACGTCTTACGGCCCTGCTGGAGGCGCCCTATAGTGAGTCGTATTA
LDV pseudoknot F	TAATACGACTCACTATAGGGCTGCTTAAGAGTTACAATGTAAGTCATGTCAGTCAGATGCAGCGACTCAGCCTTTTGTAATTAATTGCGATTTGGCTGGGCCGGAATT
LDV pseudoknot R	/i2OMeA//i2OMeA/TTCCGGCCCAGCCAAATCGCAATTAATTACAAAAGGCTGAGTCGCTGCATCTGACTGACATGACTTACATTGTAACTCTTAAGCAGCCCTATAGTGAGTCGTATTA
3pRNA F	TAATACGACTCACTATAGGGAAACTAAAAGGGAGAAGTGAAAGTG
3pRNA R	CACTTTCACTTCTCCCTTTTAGTTTCCCTATAGTGAGTCGTATTA

**Table 2:** RNA interference target sequence design.

Primer Name	Sequence
RIG-I-Forward oligo	GATCCGTAAACAACACCCATACAACGATAGTGCGTTGTATGGGTGTTGTTTACTTTTTG
RIG-I-Reverse oligo	AATTCAAAAAGTAAACAACACCCATACAACGCACTATCGTTGTATGGGTGTTGTTTACG
TLR8-Forward oligo	GATCCGAAGCTATCCTTGTGAAGACGATAGTGCGTCTTCACAAGGATAGCTTCTTTTTG
TLR8-Reverse oligo	AATTCAAAAAGAAGCTATCCTTGTGAAGACGCACTATCGTCTTCACAAGGATAGCTTCG

**Table 3:** The primers for qRT-PCR.

Primer Name	Sequence (5′ to 3′)
M-GAPDH-F	TGATGACATCAAGAAGGTGGTGAAG
M-GAPDH-R	TCCTTGGAGGCCATGTGGGCCAT
M-IFNα-F	CACACAGGCTTCCAGGCATTC
M-IFNα-R	TCTTCAGCACAAAGGACTCATCTG
M-IFNβ-F	ACGGCTCTTTCCATGAGCTAC
M-IFNβ-R	GTCAATGCAGCGTCCTCCTT
M-PKR-F	ATAGCAAGAAGGCAGAGCGTGAAG
M-PKR-R	TCAAGTCCATCCCAACAGCCATTG
M-ISG15 F	GGCAGCACAGTCCTGTTGATGG
M-ISG15 R	TGCGTCAGCCAGACCTCATAGG
M-ISG56 F	ACGTAACTGAAAATCCACAAGA
M-ISG56 R	TGCTCCAGACTATCCTTGACCT
M-RIG-I F	CTGACTGCCTCGGTTGGTGTTG
M-RIG-I R	CTTGCTCCAGTTCCTCCAGATTGTG
M-MDA5 F	AGCTTCACCTGGTGTTGGAG
M-MDA5 R	TGGCAAACTTCTTGCATGGC
M-TLR3-F	GCTCTCCTTCACCATTCC
M-TLR3-R	TTCTCAAGACCTTCCAACAT
M-TLR7-F	GGTGATGATGACAGCAAGT
M-TLR7-R	GCAACAGTCTGGTGATATTAG
M-TLR8-F	TGCTCAAGTGTTAAGTGGAA
M-TLR8-R	CAAGTCGGACAATTCAGTAAG
M-TLR9-F	GAGTGAGAACTTCCTCTACAA
M-TLR9-R	GTAATTGAAGGACAGGTTGAG
M-MAVS F	AGCCATCCCAAGGAATCCAG
M-MAVS R	GAGACAGATGGAGACACAGGC
M-TBK1-F	TGGAAGCGGCAGAGTTAGGTGAA
M-TBK1-R	TTCGGATGAGTGCCTTCTTGATGTG
M-AP-1-F	CAAGAACTCGGACCTCCT
M-AP-1-R	GTTGCTGGACTGGATTATCA
M-NEMO-F	GAGACTCAGCCTGGAGAA
M-NEMO-R	CCTTGTCCTCAGCCATCT
M-DDX3X-F	GTCCGCCACACTATGATG
M-DDX3X-R	TTCAGAGGTAGAGCCAACT
M-IKKα-F	ATACAGCGAGCAGATGAC
M-IKKα-R	CAACCTCAGCATAGTGGAT
M-IKKβ-F	AGAAGAGTGAGGTGGATATTG
M-IKKβ-R	GGACGCTGTTAAGATTATTGG
M-IKKɛ-F	GGTTGCTGTGAAGGTCTT
M-IKKɛ-R	TTGATGATGTTCTGGTGGTT
M-P50-F	CTACGCAGTGACAGGAGA
M-P50-R	GAGAATGAAGGTGGATGATTG
M-P65-F	CACTGCCGAACTCAAGAT
M-P65-R	AATACACCTCAATGTCCTCT
M-IRF3 F	CCCTTCATTGTAGGCTCCTGG
M-IRF3 R	CAGAACCAGAGGGCATAGCG
M-IRF7 F	GCAACGTGAGGGTGTGTCTT
M-IRF7 R	GCTCCATGAGCAAGCACTCAA
M-IRF9 F	GACTACTCACTGCTGCTCACCTTC
M-IRF9 R	GCTCCATGCTGCTCTCAGAACC
M-MX1-F	GCATCACCAGGGTAGCTGTA
M-MX1-R	AGATCCCGATGGTCCTGTCT
M-OAS1a F	GCGAGTTCTCCACCTGCTTCAC
M-OAS1a R	ACTAGGCGGATGAGGCTCTTGAG
M-JAK1 F	CATTAACAAGCAGGACAACA
M-JAK1 R	TAGCCATCTACCAGTGACA
M-MAPK1-F	TGGTGTGCTCTGCTTATG
M-MAPK1-R	AGTAGGTCTGGTGCTCAA
M-MAPK8-F	GTTATGGACTTGGAGGAGAG
M-MAPK8-R	ACAGACGACGATGACGAT
M-MyD88-F	CAAGTACAAGGCAATGAAGA
M-MyD88-R	CGAGTCCAGAACCAAGATT
M-STAT1-F	AGACTTCAGACCACAGACA
M-STAT1-R	CTATACTGCGTTCATCATACTG
M-STAT2-F	ACCAGCAGGATGTCTTCT
M-STAT2-R	TGTCCAGTTCATTGAGAGTT
M-STAT3-F	GTGATGCTTCCCTGATTGTG
M-STAT3-R	GCAAGGAGTGGGTCTCTAGG
M-STAT4-F	GTGTCAACCAACGATTCC
M-STAT4-R	GCTCATCACCTCCAGTAG
M-STAT5-F	AAGCAGCAGACCATCATC
M-STAT5-R	CTTCTCACACCAGGACTG
M-STAT6-F	CAGCGACTCAGAGATTGG
M-STAT6 R	GAACGAATGGACAGGTCTT
M-TYK2-F	CCTGAGTCTGCTGCTAGA
M-TYK2-R	TTGTTCACCTCCTCCTGTA

## Data Availability

All data for these studies are contained within this article.
